# Autochthonous and Dormant *Cryptococcus gattii* Infections in Europe

**DOI:** 10.3201/eid1810.120068

**Published:** 2012-10

**Authors:** Ferry Hagen, M. Francisca Colom, Daniëlle Swinne, Kathrin Tintelnot, Roberta Iatta, Maria Teresa Montagna, Josep M. Torres-Rodriguez, Massimo Cogliati, Aristea Velegraki, Arjan Burggraaf, Alwin Kamermans, Johanna M. Sweere, Jacques F. Meis, Corné H.W. Klaassen, Teun Boekhout

**Affiliations:** Royal Netherlands Academy of Arts and Sciences Fungal Biodiversity Centre, Utrecht, the Netherlands (F. Hagen, A. Burggraaf, A. Kamermans, J.M. Sweere, T. Boekhout);; University Medical Center Utrecht, Utrecht (F. Hagen, T Boekhout);; Universidad Miguel Hernàndez, Alicante, Spain (M.F. Colom); Institute of Public Health, Brussels, Belgium (D. Swinne);; Robert Koch-Institut, Berlin, Germany (K. Tintelnot); Università degli Studi di Bari, Bari, Italy (R. Iatta, M.T. Montagna);; Autonomous University of Barcelona, Barcelona, Spain (J.M. Torres-Rodriguez);; Università degli Studi di Milano, Milan, Italy (M. Cogliati);; University of Athens, Athens, Greece (A. Velegraki);; University College Utrecht, Utrecht (J.M. Sweere);; Radboud University Nijmegen Medical Centre, Nijmegen, the Netherlands (J.F. Meis);; and Canisius Wilhelmina Hospital, Nijmegen (J.F. Meis, C.H.W. Klaassen)

**Keywords:** Cryptococcus gattii, multilocus sequence typing, amplified fragment length polymorphism analysis, epidemiology, genotyping, Europe, yeast, fungi

## Abstract

Until recently, *Cryptococcus gattii* infections occurred mainly in tropical and subtropical climate zones. However, during the past decade, *C. gattii* infections in humans and animals in Europe have increased. To determine whether the infections in Europe were acquired from an autochthonous source or associated with travel, we used multilocus sequence typing to compare 100 isolates from Europe (57 from 40 human patients, 22 from the environment, and 21 from animals) with 191 isolates from around the world. Of the 57 human patient isolates, 47 (83%) were obtained since 1995. Among the 40 patients, 24 (60%) probably acquired the *C. gattii* infection outside Europe; the remaining 16 (40%) probably acquired the infection within Europe. Human patient isolates from Mediterranean Europe clustered into a distinct genotype with animal and environmental isolates. These results indicate that reactivation of dormant *C. gattii* infections can occur many years after the infectious agent was acquired elsewhere.

## Abstract 2

Dormant infections can become reactivated years after having been acquired on another continent.

During the past decade, the basidiomycetous yeast *Cryptococcus gattii* has stepped out of the shadows of its sibling *C. neoformans*. The latter species mainly infects immunocompromised persons, and *C. gattii* mainly infects apparently immunocompetent persons. *C. neoformans* is found globally, and *C. gattii* has been mostly limited to tropical and subtropical areas in Central Africa, northern Australia, and Central and South America (*1*). However, this distribution pattern changed after an unprecedented outbreak of *C. gattii* emerged in the temperate climate of British Columbia, Canada, and expanded to the Pacific Northwest region of Canada and the United States (*1*,*2*). Epidemiologic studies have shown that *C. gattii* occurs in areas other than tropical or subtropical zones, such as in Mediterranean Europe, northern Europe, and western Australia (*3*–*5*).

For the purpose of studying the epidemiology of *C. gattii*, a broad variety of molecular biological techniques have been developed, including PCR fingerprinting, restriction fragment length polymorphism analysis of the *PLB1* and *URA5* loci, amplified fragment length polymorphism (AFLP) fingerprint analysis, and several multilocus sequence typing (MLST) approaches (*6*–*9*). These laboratory investigations have shown that *C. gattii* can be divided into 5 distinct genotypes: AFLP4/VGI, AFLP5/VGIII, AFLP6/VGII, AFLP7/VGIV, and AFLP10/VGIV (*8*,*9*). Serotype B strains occur in genotypes AFLP4/VGI, AFLP6/VGII, and AFLP10/VGIV; serotype C strains are restricted to genotypes AFLP5/VGIII and AFLP7/VGIV (*8*).

Recently, a consensus MLST scheme was proposed for epidemiologic investigations of *C. gattii* and *C. neoformans*, specifically, the nuclear loci *CAP59*, *GPD1*, IGS1, *LAC1*, *PLB1*, *SOD1*, and *URA5* (*9*). So far, this consensus MLST scheme has been used to study the population structure of *C. neoformans* strains from Thailand and *C. gattii* strains from Australia (*3*,*10*).

We investigated the occurrence of *C. gattii* in Europe, focusing on whether this pathogen is emerging and, if so, how to explain this emergence pattern. Furthermore, we explored whether the infections originated from Europe or were introduced from other continents. To achieve these goals, members of the European Confederation of Medical Mycology were asked to send recently obtained human patient isolates of the species for detailed AFLP and MLST analyses. Thus, the genetic diversity of the yeast was used to trace its geographic origin to identify where the infections were acquired. AFLP genotyping results were combined with published *C. gattii* MLST results from Byrnes et al. (*11*) and Fraser et al. (*12*), which were extended to match the *Cryptococcus* consensus MLST scheme (*9*). Our study produced the following 5 conclusions: all hitherto known genotypes of *C. gattii* are emerging in Europe; genotype AFLP4/VGI isolates predominate; a *C. gattii* cluster, which is endemic to Mediterranean Europe and genetically distinct from the other populations, exists; several human infections are caused by travel-related acquisition of *C. gattii* outside Europe; and autochthonous cases occur in Europe.

## Materials and Methods

### Strains and Media

We compared a collection of 107 isolates collected from Europe with 194 isolates collected globally (Table 1; Technical Appendix Table 1). All isolates were checked for purity and cultivated on malt extract agar medium (Oxoid, Basingstoke, UK). Cultures were incubated for 2 days at 30°C. A working collection was made by growing *C. gattii* strains on malt extract agar slants for 2 days at 30°C, after which the strains were stored at 4°C. Strains were stored long term at −80°C by using the Microbank system (Pro-Lab Diagnostics, Richmond Hill, Ontario, Canada).

**Table:** Distribution of *Cryptococcus gattii* strains*

Source of isolation	Genotype					Total†	Total (%)	
	AFLP4/VGI	AFLP5/VGIII	AFLP6/VGII	AFLP7/VGIV	AFLP10/VGIV			
All *C. gattii* isolates	146	22	108	13	2	0	291 (100)
Human	84	16	68	12	2	0	182 (62.5)‡	
Environment	37	5	17	0	0	0	59 (20.3)‡	
Animal	24	0	23	1	0	0	48 (16.5)‡	
Unknown	1	1	0	0	0	0	2 (0.7)‡	
Africa								
Human	18	0	3	8	0	29	36 (12.7)§	
Environment	6	0	0	0	0	6		
Animal	0	0	0	1	0	1		
Asia (clinical)	19	0	5	2	0	26	26 (8.9)§	
Australia								
Human	2	1	8	0	0	11	18 (6.2)§	
Environment	5	0	1	0	0	6		
Animal	0	0	1	0	0	1		
Europe¶								
Human	29#	1**	23††	2‡‡	2§§	57	100 (34.4)§	
Environment	22	0	0	0	0	22		
Animal	21	0	0	0	0	21		
North America¶								
Human	3	8	19	0	0	30	69 (23.7)§	
Environment	4	3	10	0	0	17		
Animal	2	0	20	0	0	22		
South America¶								
Human	12	3	10	0	0	25	36 (12.4)§	
Environment	0	2	6	0	0	8		
Animal	1	0	2	0	0	3		
Unknown								
Human	1	3	0	0	0	4	6 (2.1)§	
Unknown	1	1	0	0	0	2		

*The number of *C. gattii* isolates is provided for each geographic area and further subdivided per genotype and source of isolation. AFLP, amplified fragment length polymorphism; human, human clinical patient. †Total number of isolates per geographic area. ‡The percentage of isolates is given for the source of isolation subset. §The percentage of isolates is given as a percentage of all isolates. ¶Ten interspecies hybrid *C. gattii* × *C. neoformans* isolates were excluded from the set of isolates from Europe (n = 7), North America (n = 1), and South America (n = 2). #Twelve patients with an autochthonous acquired infection (16 isolates) and 11 patients with an infection acquired outside Europe (13 isolates). **Infection acquired outside Europe. ††Four patients with an autochthonous infection (10 isolates) and 10 patients with an infection acquired outside Europe (13 isolates). ‡‡Two phenotypically different isolates from an emigrant from Zambia. §§Two phenotypically different isolates from an emigrant from Mexico.

### Amplification and Sequencing of MLST Loci

Genomic DNA extraction, AFLP genotyping, and mating-type determination by amplification of either the *STE12***a** or *STE12*α locus were performed as described (*8*). The 7 nuclear consensus MLST loci (*CAP59*, *GPD1*, IGS1, *LAC1*, *PLB1*, *SOD1*, and *URA5*) were amplified by using the preferred primer combinations (*9*). To compare the current set of *C. gattii* strains with those from a published *C. gattii* population biology study, we included the 3 nuclear loci that are not part of the consensus MLST scheme (*CAP10*, *MPD1*, and *TEF1*α) (*12*). We also included isolates from a global study by Byrnes et al. (*11*) and Fraser et al. (*12*) and subjected them to amplification and sequencing of the *CAP59*, *SOD1*, and *URA5* loci.

Amplifications were conducted in a 25-µL PCR mixture containing 37.5 mmol/L MgCl_2_ (Bioline, London, UK), 1× PCR buffer (Bioline), 1.9 mmol/L dNTPs (Bioline), 0.5 U Taq DNA polymerase (Bioline), 5 pmol of both primers (Biolegio, Nijmegen, the Netherlands) (Technical Appendix Table 2), and ≈100 ng of genomic DNA. PCRs were conducted with an initial denaturation step at 94°C for 5 min, followed by 35 cycles of denaturation at 94°C for 30s, annealing for 30s (see Technical Appendix Table 2 for optimal annealing temperatures), extension at 72°C for 1 min, followed by 72°C for 5 min and a final dwell at 21°C.

Sequencing reactions were conducted with the BigDye version 3.1 chemistry kit (Applied Biosystems, Foster City, CA, USA) as described (*13*). For all amplification products except *CAP59*, the initial amplification primers were used for sequencing reactions. For *CAP59*, the newly designed forward primer CAP59L-Fwd and the original reverse primer JOHE15438 (*12*) were used.

### Sequence Alignment and Phylogenetic and Recombination Analyses

Consensus sequences were assembled and checked for ambiguities by using SeqMan version 8.0.2 (DNASTAR, Madison, WI, USA). Sequence alignments were generated with MEGA version 5 (*14*) by using the standard settings and manual correction. The genome sequence databases of reference strains H99 (culture collection no. CBS8710; *C. neoformans* variety *grubii*; AFLP1/VNI; Broad Institute [www.broadinstitute.org/annotation/genome/cryptococcus_neoformans/]) and JEC21 (culture collection no. CBS10513; *C. neoformans* variety *neoformans*; AFLP2/VNIV; Stanford Genome Technology Center [www-sequence.stanford.edu/group/C.neoformans/]) were used to extract the corresponding sequences for all 10 investigated nuclear loci to serve as an outgroup. The best fitting nucleotide substitution model was determined by using MrModeltest version 2 (*15*) and was conducted for the complete *C. gattii* 10-loci MLST, for the accepted consensus MLST scheme (*CAP59*, *GPD1*, IGS1, *LAC1*, *PLB1*, *SOD1*, and *URA5*) (*9*), and a previously launched *C. gattii* MLST scheme (*CAP10*, *GPD1*, IGS1, *LAC1*, *MPD1*, *PLB1*, and *TEF1*) (*12*). As a result, the HKY G+I model (Hasegawa-Kishino-Yano plus gamma distributed with invariant sites) was the best model to use for analyzing the phylogeny of the *C. gattii* isolates for all 3 datasets. The evolutionary history was inferred by using the maximum-likelihood method in MEGA version 5 (*14*). A bootstrap consensus tree was inferred from 1,000 replicates to show the relevant lineages obtained in this analysis.

We calculated the haplotype diversity (*H_R_*), equal to the Simpson diversity index (*D*), by using the Microsoft Excel (Microsoft, Redmond, WA, USA) add-in called Haplotype Analysis (*16*). For this purpose, sequences were collapsed into sequence type numbers (Technical Appendix Table 1).

## Results

### AFLP Genotypes and Geographic Distribution

The 301 *C. gattii* isolates collected from Europe and other areas around the world could be divided into the following genotypes: 146 AFLP4/VGI (50.2%; 72 from Europe), 22 AFLP5/VGIII (7.6%; 1 from Europe), 108 AFLP6/VGII (37.1%; 23 from Europe), 13 AFLP7/VGIV (4.5%; 2 from Europe), and 2 AFLP10/VGIV (0.7%; both from Europe). From 10 isolates (7 AFLP8 [5 from Europe] and 3 AFLP9 [2 from Europe]), genotypes represented interspecies *C. neoformans* × *C. gattii* hybrids. These 10 isolates were excluded from further analysis because amplified fragments of hybrid isolates will result in mixtures of different alleles. The 57 human patient isolates from Europe were obtained from 40 patients (Technical Appendix Table 1). The genotypic diversity of the remaining 291 *C. gattii* isolates (100 from Europe, 191 from other areas) with a haploid genotype is shown in the Table.

### Phylogeographic Origin

Sequence type diversity was calculated for each of the MLST loci, all 10 loci, and the combined datasets according to Fraser et al. (*12*) and Meyer et al. (*9*) (Technical Appendix Table 3). The *CAP10* locus showed the lowest overall diversity (n_ST_ = 19; *D*_ST_ = 0.765) and the IGS1 locus showed the highest diversity (n_ST_ = 52; *D*_ST_ = 0.930). The 10-loci MLST dataset showed the highest diversity (n_ST_ = 150; *D*_ST_ = 0.975), followed by the MLST scheme of Meyer et al. (*D*_ST_ = 0.971) (*9*) and Fraser et al. (*D*_ST_ of 0.959) (*12*). The latter MLST scheme differentiated more sequence types than the consensus MLST scheme (n_ST_ = 136 vs. 127).

Maximum-likelihood analysis of the MLST data showed that the *C. gattii* isolates clustered in 5 monophyletic clusters, which were highly supported and agreed with the AFLP genotypes (Figure, Technical Appendix Figure). When each genotypic cluster was separately analyzed, support values of the branches were low, <75 (Technical Appendix Figure). For genotype AFLP4/VGI isolates, bootstrap support values for nearly all branches were low; for the second largest group formed by AFLP6/VGII isolates, branches were better supported.

**Figure:**
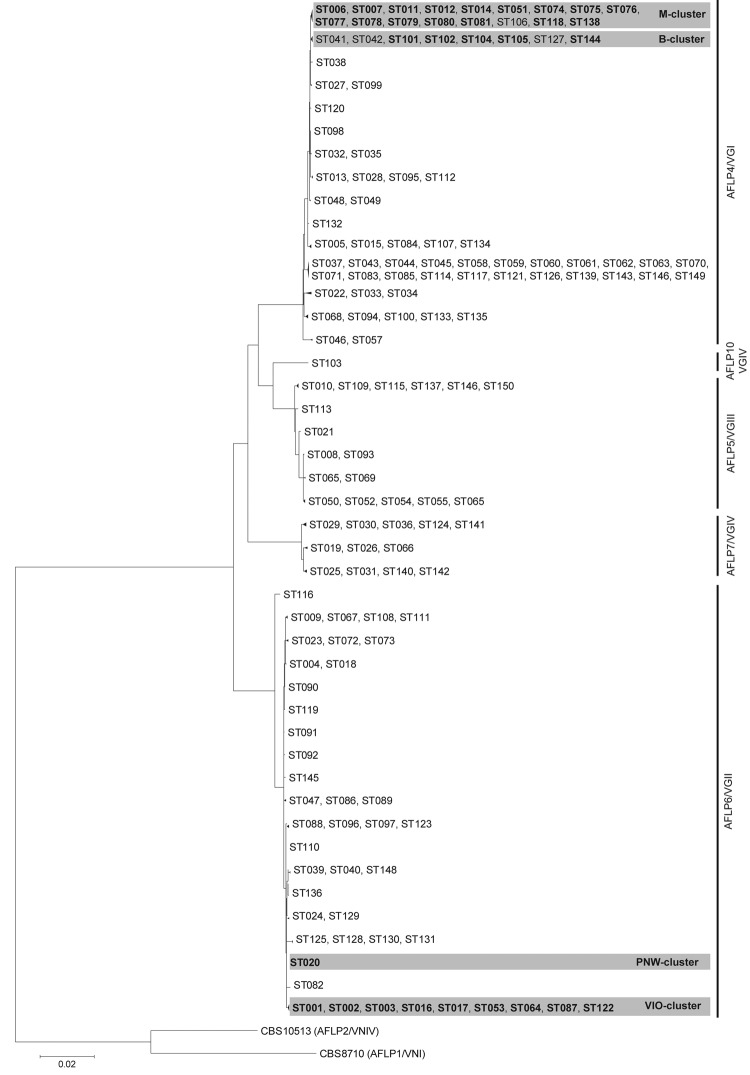
Maximum-likelihood phylogenetic analysis based on 10-loci multilocus sequence type data of *Cryptococcus gattii* isolates (condensed). Phylogenetic relatedness of 150 STs representing the 291 *C. gattii* isolates, calculated by using the maximum-likelihood algorithm and rooted by using the 2 *C. neoformans* reference strains CBS8710 (genotype AFLP1/VNI) and CBS10513 (genotype AFLP2/VNIV). Closely related sequence types were collapsed into 1 branch shown by multiple sequence type numbers. **Boldface** indicates sequence types that are within a shaded area belong to a specified *C. gattii* cluster; B, M, PNW, and VIO represent clusters from Brazil; Mediterranean Europe; the US Pacific Northwest outbreak; and the Vancouver Island, British Columbia, Canada, outbreak, respectively. AFLP, amplified fragment length polymorphism; ST, sequence type. Scale bar indicates number of substitutions per site. See Technical Appendix Figure for a detailed phylogenetic analysis.

Phylogenetic analysis demonstrated that several human patient genotype AFLP4/VGI isolates from Europe had an autochthonous origin because they formed a separate cluster with environmental and animal isolates from the same area. A set of isolates from human patients on the Iberian Peninsula (CCA232, CCA242L, CCA242T, CCA311, CCA312, CL6148) were found to be genetically indistinguishable from isolates obtained from animals or the environment in the same area (indicated in Technical Appendix Figure as the European Mediterranean cluster). The human patient isolates from Europe with genotype AFLP4/VGI, which were probably acquired within Europe because patients did not have histories of travel outside Europe, are IP2005/215 and IP2006/958 (France), RKI85/888 (Germany), 5UM and 75UM (Italy), RKI97/482 (Portugal), and CBS2502 (the Netherlands). Some of these human patient isolates were closely related (e.g., IP2005/215 and RKI97/482) or even genetically indistinguishable (e.g., CBS2502 and RKI85/888).

A large proportion of the human patient and environmental isolates of *C. gattii* AFLP4/VGI from Italy and Spain formed a novel autochthonous Mediterranean MLST cluster that was genetically homogeneous, irrespective of their origin or mating type (Figure, Technical Appendix Figure). *C. gattii* AFLP4/VGI was involved with numerous small outbreaks among goats in Spain, and the isolates were genetically indistinguishable from recently obtained human patient, animal, and environmental isolates from different provinces in Spain as well as from AFLP4/VGI mating-type **a** isolates from Italy (Technical Appendix Figure).

Several *C. gattii* genotype AFLP6/VGII infections were found to have originated in Europe (e.g., the IP1998/1037–1 and −2, IP2003/125, and CCA242 isolates), and all fell within a cluster that could not be linked to an environmental source. The same holds true for the human patient isolates from Greece (AV54S, –W, and IUM01–4731), which came from the same patient who had no history of travel outside Greece.

In the phylogenetic analysis, isolates from human patients in Europe were also observed next to those originating from other geographic areas. The most striking example was a set of 4 human patient isolates from citizens of Denmark, the Netherlands, Germany, and Switzerland, in whom cryptococcosis developed after they had visited Vancouver Island, British Columbia, Canada. The isolates obtained from these tourists (CBS10485, RKI06/496, RKI01/774, and CBS10866, respectively) had MLST profiles identical to that of *C. gattii* AFLP6A/VGIIa, the genotype that caused the Vancouver Island outbreak (Figure, Technical Appendix Figure) (*17*–*19*). The outbreak-related sets of AFLP6A/VGIIa and AFLP6C/VGIIc isolates from the Vancouver Island and Pacific Northwest outbreaks, respectively, are within outbreak-specific clusters (Figure, Technical Appendix Figure). A similar finding was observed for a set of 5 human patient *C. gattii* AFLP6B/VGIIb isolates (IP1996/1120–1 and −2, IP1999/901–1 and −2, and IP2000/87) in France, which had been obtained from patients who had emigrated from Africa to France and which were genetically indistinguishable from an isolate (IP2001/935–1) from a resident of Senegal (Technical Appendix Figure). This set of 5 isolates from France and 1 from Senegal were closely related to isolates from the Vancouver Island and Pacific Northwest outbreaks; however, it seems unlikely that the patient from Senegal had traveled to these outbreak areas, and none of the patients in France reported having traveled to Canada or the United States.

A set of *C. gattii* AFLP4/VGI mating-type α isolates from Portugal (IP1997/18) and Belgium (IHEM19725B and –S) was found to be indistinguishable from human patient isolates from the Democratic Republic of the Congo and Rwanda (B3939 and CBS6289, which were both mating-type **a**, and IHEM10602S, IHEM10769S, and IHEM10769W, which were mating-type α) (Figure, Technical Appendix Figure). Both phenotypically different isolates of IHEM19725 were obtained from an HIV-infected patient from Rwanda who had emigrated to Belgium. The CBS1622 isolate from Europe, obtained from a patient with the oldest documented case of a *C. gattii* infection (*20*), was found to be genetically indistinguishable from a set of isolates from North America.

The single *C. gattii* AFLP5/VGIII isolate from a 24-year-old immunocompetent patient from Germany (isolate RKI97/310) (*21*) clustered with human patient and environmental isolates from Mexico (Technical Appendix Figure). The 2 *C. gattii* AFLP7/VGIV isolates CBS7952D and CBS7952S, obtained from an HIV-infected patient in Sweden, were genetically indistinguishable from each other and had a unique MLST profile that clustered with human patient isolates from Africa. According to the patient’s history, years before the onset of cryptococcal infection, she had emigrated from Zambia to Sweden (Technical Appendix Figure). Another example of a reactivated dormant *C. gattii* infection is that of the human patient isolate from Italy, IUM92–6682 (AFLP4/VGI), obtained from an immunocompetent immigrant from Brazil, which had an identical MLST profile to 6 human patient mating-type α isolates (IHEM14934, IHEM14956, IHEM14965, IHEM14968, IHEM14976, and IHEM14984) from Brazil (Figure, Technical Appendix Figure).

Infection was acquired outside Europe for 24 of these patients (31 isolates) and within Europe for 16 patients (26 isolates) (Table, Technical Appendix Figure). Among these 57 human patient isolates, most (47 [82.5%]) were obtained since 1995, the remaining 10 (17.5%) were isolated during 1895–1994. One of these isolates originated from 1985, another 2 isolates (CBS1622 and CBS2502), from 1895 and 1957, were retrospectively found to represent *C. gattii* isolates from Europe (*20*,*22*).

## Discussion

In Europe, *C. gattii* has been reported as a rare cause of apparently autochthonous cryptococcal infections (*1*). The earliest documented case that turned out to be caused by *C. gattii* in Europe was made by Curtis in 1896 (*20*). Until the 1980s, cryptococcosis was rarely observed in Europe and *C. gattii* infections were especially rare; only 2 cases have retrospectively been found (*20*,*22*). In the 1980s, infections were reported for 2 immunocompetent citizens of Germany, who had never traveled abroad (*23*,*24*). Subsequent case reports described *C. gattii* infections that were imported or acquired in Europe (*25*,*26*). Since 1995, the number of reports of *C. gattii* infections increased and describe *C. gattii* infections in humans and animals from Greece (*27*), Italy (*28*,*29*), and Spain (*5*,*30*–*33*). In the current study, 8 (20%) cases were observed until 1995, and 32 (80%) cases in humans have been observed since 1995. We excluded cases for which an isolate was not available for confirmation. This exclusion is especially relevant in that we re-identified a reported *C. gattii* infection as actually being caused by *C. neoformans* (*34*). These data suggest that during the past 2 decades, *C. gattii* has been emerging in Europe.

In the current study, 16 (40%) of the human patient isolates from Europe were found to have an autochthonous origin in Europe that either could be linked to environmental isolates from the Mediterranean area or that came from patients who had never traveled outside their resident country. A total of 24 (60%) isolates could be linked to *C. gattii*–endemic regions in Brazil, the United States, Africa, and the Vancouver Island outbreak region. These observations demonstrate that *C. gattii* infections can be imported subclinically and can cause infections after being dormant for many years.

Nearly all isolates from Mediterranean Europe belonged to genotype AFLP4/VGI, and MLST analysis showed that these isolates form a separate cluster within this genotype (Figure, Technical Appendix Figure) (*5*). *C. gattii* genotype AFLP4/VGI has also recently been reported from the environment in the Netherlands, but these isolates were not similar to any of the AFLP4/VGI isolates from Mediterranean Europe or to isolate CBS2502, which was isolated in 1957 from a pregnant citizen of the Netherlands, who had never traveled abroad but who died of cryptococcosis (*4*). Isolate CBS2502 is genotypically identical to isolate RKI85/888, which was isolated from a previously healthy citizen of Germany, who also had never traveled outside Germany. These 2 cases strongly suggest that different genotypes of *C. gattii* AFLP4/VGI occur in the environment of northwestern Europe because isolates from patients were genetically different from the recently reported isolates from the environment of the Netherlands (this study; *4*).

In conclusion, *C. gattii* is emerging in Europe, and the isolates from Europe can be divided into 5 genotypic clusters. Most *C. gattii* infections in Europe are probably autochthonous, and several infections are proven to have been acquired outside the European continent, e.g., during visits to *C. gattii*–endemic regions, such as the Vancouver Island outbreak area or before migration to Europe from *C. gattii*–endemic regions in Africa and South America. Reactivation of dormant *C. gattii* infections and of infections acquired outside Europe after immune suppression occurs more often than previously assumed. This finding might suggest that *C. gattii* infections caused by certain genotypes are associated with altered immune status of the human host. Thus, *C. gattii* is probably a more opportunistic pathogen, as has recently been hypothesized, than a strictly primary pathogen (*18*,*35*). *C. gattii* isolates with genotypes AFLP5/VGIII and AFLP7/VGIV were rarely found in Europe and were all acquired outside the European continent. However, these genotypes have frequently been isolated from HIV-infected persons and other immunocompromised patients in Africa and the American continents (*1*,*7*,*36*). A connection seems to exist between these *C. gattii* genotypes and the host’s immune status. Further epidemiologic and immunologic research is needed to unravel this apparent correlation.

Technical Appendix Detailed maximum-likelihood phylogenetic analysis based on 10-loci multilocus sequence typing data of *Cryptococcus gattii* isolates, background information of *Cryptococcus gattii* isolates and GenBank accession numbers for the 10-loci multilocus sequence typing data, primers used for multilocus sequence typing, and sequence type diversity for each of the investigated loci and amplified fragment length polymorphism genotype clusters.

## References

[1] Springer DJ, Chaturvedi V. Projecting global occurrence of *Cryptococcus gattii.* Emerg Infect Dis. 2010;16:14–20. 10.3201/eid1601.090369 20031037 PMC2874352

[2] Kidd SE, Hagen F, Tscharke RL, Huynh M, Bartlett KH, Fyfe M, A rare genotype of *Cryptococcus gattii* caused the cryptococcosis outbreak on Vancouver Island (British Columbia, Canada). Proc Natl Acad Sci U S A. 2004;101:17258–63. 10.1073/pnas.0402981101 15572442 PMC535360

[3] Carriconde F, Gilgado F, Arthur I, Ellis D, Malik R, van de Wiele N, Clonality and α-a recombination in the Australian *Cryptococcus gattii* VGII population—an emerging outbreak in Australia. PLoS One. 2011;6:e16936. 10.1371/journal.pone.0016936 21383989 PMC3044715

[4] Chowdhary A, Randhawa HS, Boekhout T, Hagen F, Klaassen CHW, Meis JF. Temperate climate niche for *Cryptococcus gattii* in northern Europe. Emerg Infect Dis. 2012;18:172–4. 10.3201/eid1801.111190 22261398 PMC3310122

[5] Colom MF, Hagen F, Gonzalez A, Mellado A, Morera N, Linares C, *Ceratonia siliqua* (carob) trees as natural habitat and source of infection by *Cryptococcus gattii* in the Mediterranean environment. Med Mycol. 2012;50:67–73. 21521012 10.3109/13693786.2011.574239

[6] Boekhout T, Theelen B, Diaz M, Fell JW, Hop WC, Abeln EC, Hybrid genotypes in the pathogenic yeast *Cryptococcus neoformans.* Microbiology. 2001;147:891–907. 11283285 10.1099/00221287-147-4-891

[7] Bovers M, Hagen F, Boekhout T. Diversity of the *Cryptococcus neoformans*–*Cryptococcus gattii* species complex. Rev Iberoam Micol. 2008;25:S4–12. 10.1016/S1130-1406(08)70019-6 18338917

[8] Hagen F, Illnait-Zaragozi MT, Bartlett KH, Swinne D, Geertsen E, Klaassen CH, In vitro antifungal susceptibilities and amplified fragment length polymorphism genotyping of a worldwide collection of 350 clinical, veterinary, and environmental *Cryptococcus gattii* isolates. Antimicrob Agents Chemother. 2010;54:5139–45. 10.1128/AAC.00746-10 20855729 PMC2981230

[9] Meyer W, Aanensen DM, Boekhout T, Cogliati M, Diaz MR, Esposto MC, Consensus multi-locus sequence typing scheme for *Cryptococcus neoformans* and *Cryptococcus gattii.* Med Mycol. 2009;47:561–70. 10.1080/13693780902953886 19462334 PMC2884100

[10] Simwami SP, Khayhan K, Henk DA, Aanensen DM, Boekhout T, Hagen F, Low diversity *Cryptococcus neoformans* variety *grubii* multilocus sequence types from Thailand are consistent with an ancestral African origin. PLoS Pathog. 2011;7:e1001343. 10.1371/journal.ppat.1001343 21573144 PMC3089418

[11] Byrnes EJ III, Li W, Lewit Y, Ma H, Voelz K, Ren P, Emergence and pathogenicity of highly virulent *Cryptococcus gattii* genotypes in the northwest United States. PLoS Pathog. 2010;6:e1000850. 10.1371/journal.ppat.1000850 20421942 PMC2858702

[12] Fraser JA, Giles SS, Wenink EC, Geunes-Boyer SG, Wright JR, Diezmann S, Same-sex mating and the origin of the Vancouver Island *Cryptococcus gattii* outbreak. Nature. 2005;437:1360–4. 10.1038/nature04220 16222245

[13] Bovers M, Hagen F, Kuramae EE, Boekhout T. Six monophyletic lineages identified within *Cryptococcus neoformans* and *Cryptococcus gattii* by multi-locus sequence typing. Fungal Genet Biol. 2008;45:400–21. 10.1016/j.fgb.2007.12.004 18261945

[14] Tamura K, Peterson D, Peterson N, Stecher G, Nei M, Kumar S. MEGA5: Molecular Evolutionary Genetics Analysis using maximum likelihood, evolutionary distance, and maximum parsimony methods. Mol Biol Evol. 2011;28:2731–9. 10.1093/molbev/msr121 21546353 PMC3203626

[15] Nylander JAA. MrModeltest v.2. Uppsala: Evolutionary Biology Centre, Uppsala University: 2004 [cited 2012 Aug 9]. http://www.abc.se/~nylander/

[16] Eliades N-G, Eliades DG. Haplotype Analysis: software for analysis of haplotype data. Forest Goettingen (Germany): Genetics and Forest Tree Breeding, Georg-August University Goettingen,. 2009 [cited 2012 Aug 9]. http://www.uni-goettingen.de/en/134935.html

[17] Georgi A, Schneemann M, Tintelnot K, Calligaris-Maibach RC, Meyer S, Weber R, *Cryptococcus gattii* meningoencephalitis in an immunocompetent person 13 months after exposure. Infection. 2009;37:370–3. 10.1007/s15010-008-8211-z 19390780

[18] Hagen F, van Assen S, Luijckx GJ, Boekhout T, Kampinga GA. Activated dormant *Cryptococcus gattii* infection in a Dutch tourist who visited Vancouver Island (Canada): a molecular epidemiological approach. Med Mycol. 2010;48:528–31. 10.3109/13693780903300319 19824880

[19] Lindberg J, Hagen F, Laursen A, Stenderup J, Boekhout T. *Cryptococcus gattii* risk for tourists visiting Vancouver Island, Canada. Emerg Infect Dis. 2007;13:178–9. 10.3201/eid1301.060945 17370544 PMC2725802

[20] Curtis F. Contribution a l’étude de la saccharomycose humaine. Ann Inst Pasteur (Paris). 1896;10:449–68.

[21] Grosse P, Tintelnot K, Söllner O, Schmitz B. Encephalomyelitis due to *Cryptococcus neoformans* var. *gattii* presenting as spinal tumour: case report and review of the literature. J Neurol Neurosurg Psychiatry. 2001;70:113–6. 10.1136/jnnp.70.1.113 11118259 PMC1763492

[22] Janssens J, Beetstra A. Torulosis in a pregnant female, with localization in the lungs [in Dutch]. Ned Tijdschr Geneeskd. 1957;101:824–6. 13451717

[23] Kohl KH, Hof H, Schrettenbrunner A, Seeliger HP, Kwon-Chung KJ. *Cryptococcus neoformans* var. *gattii* in Europe. Lancet. 1985;325:1515. 10.1016/S0140-6736(85)92300-1 2861448

[24] Schaberg T, Mai J, Thalmann U, Seibold M, Staib F. Cryptococcoma of the lung—a contribution to diagnosis and therapy [in German]. Internist (Berl). 1988;29:510–5. 3049426

[25] Dromer F, Ronin O, Dupont B. Isolation of *Cryptococcus neoformans* var. *gattii* from an Asian patient in France: evidence for dormant infection in healthy subjects. J Med Vet Mycol. 1992;30:395–7. 10.1080/02681219280000511 1469541

[26] Dromer F, Mathoulin S, Dupont B, Laporte A. Epidemiology of cryptococcosis in France: a 9-year survey (1985–1993). French Cryptococcosis Study Group. Clin Infect Dis. 1996;23:82–90. 10.1093/clinids/23.1.82 8816134

[27] Velegraki A, Kiosses VG, Pitsouni H, Toukas D, Daniilidis VD, Legakis NJ. First report of *Cryptococcus neoformans* var. *gattii* serotype B from Greece. Med Mycol. 2001;39:419–22. 12054052 10.1080/mmy.39.5.419.422

[28] Iatta R, Hagen F, Fico C, Lopatriello N, Boekhout T, Montagna MT. *Cryptococcus gattii* infection in an immunocompetent patient from southern Italy. Mycopathologia. 2012 ;174:87–92. 10.1007/s11046-011-9493-8 22057831

[29] Montagna MT, Viviani MA, Pulito A, Aralla C, Tortorano AM, Fiore L, *Cryptococcus neoformans* var. *gattii* in Italy. Note II. Environmental investigation related to an autochthonous clinical case in Apulia. J Mycol Med. 1997;7:93–6.

[30] Baró T, Torres-Rodríguez JM, De Mendoza MH, Morera Y, Alía C. First identification of autochthonous *Cryptococcus neoformans* var. *gattii* isloated from goats with predominantly severe pulmonary disease in Spain. J Clin Microbiol. 1998;36:458–61. 9466758 10.1128/jcm.36.2.458-461.1998 PMC104559

[31] Colom MF, Frasés S, Ferrer C, Jover A, Andreu M, Reus S, First case of human cryptococcosis due to *Cryptococcus neoformans* var. *gattii* in Spain. J Clin Microbiol. 2005;43:3548–50. 10.1128/JCM.43.7.3548-3550.2005 16000503 PMC1169187

[32] Guinea J, Hagen F, Peláez T, Boekhout T, Tahoune H, Torres-Narbona M, Antifungal susceptibility, serotyping, and genotyping of clinical *Cryptococcus neoformans* isolates collected during 18 years in a single institution in Madrid, Spain. Med Mycol. 2010;48:942–8. 10.3109/13693781003690067 20297948

[33] Solla I, Morano LE, Vasallo F, Cuenca-Estrella M. *Cryptococcus gattii* meningitis: observation in a Spanish patient [in Spanish]. Enferm Infecc Microbiol Clin. 2008;26:395–6. 10.1157/13123846 18588823

[34] Bodasing N, Seaton RA, Shankland GS, Kennedy D. Cryptococcus *neoformans* var. *gattii* meningitis in an HIV-positive patient: first observation in the United Kingdom. J Infect. 2004;49:253–5. 10.1016/j.jinf.2003.06.001 15337344

[35] MacDougall L, Fyfe M, Romney M, Starr M, Galanis E. Risk factors for *Cryptococcus gattii* infection, British Columbia, Canada. Emerg Infect Dis. 2011;17:193–9. 10.3201/eid1702.101020 21291588 PMC3204768

[36] Litvintseva AP, Thakur R, Reller LB, Mitchell TG. Prevalence of clinical isolates of *Cryptococcus gattii* serotype C among patients with AIDS in sub-Saharan Africa. J Infect Dis. 2005;192:888–92. 10.1086/432486 16088839

