# Supplementary material for: Autochthonous and Dormant Cryptococcus gattii Infections in Europe
Source: Emerg Infect Dis. 2012 Oct;18(10):1618–24. doi: 10.3201/eid1810.120068 (PMC3471617; doi:10.3201/eid1810.120068)
Supplement: Technical Appendix — Detailed maximum-likelihood phylogenetic analysis based on 10-loci multilocus sequence typing data of Cryptococcus gattii isolates, background information of Cryptococcus gattii isolates and GenBank accession numbers for the 10-loci multilocus sequence typing data, primers used for multilocus sequence typing, and sequence type diversity for each of the investigated loci and amplified fragment length polymorphism genotype clusters. [file 12-0068-Techapp-s1.pdf]

Article DOI: http://dx.doi.org/10.3201/eid1810.120068

Autochthonous and Dormant *Cryptococcus gattii* Infections in Europe

Technical Appendix

Figure (see last page). Maximum-likelihood phylogenetic analysis based on 10-loci multilocus sequence typing data of *Cryptococcus gattii* isolates (detailed). Phylogenetic relatedness of 291 *C. gattii* isolates calculated by using the maximum-likelihood algorithm with 1,000 bootstraps and rooted by using the 2 *C. neoformans* reference strains CBS8710 (genotype AFLP1/VNI) and CBS10513 (genotype AFLP2/VNIV). The 5 *C. gattii* AFLP genotypes AFLP4/VGI, AFLP5/VGIII, AFLP6/VGII, AFLP7/VGIV, and AFLP10/VGIV are highly supported with bootstrap values of ≥75, as indicated next to the branches. Isolate numbers that are within a shaded area belong to a specified *C.* gattii cluster. Amplified fragment length polymorphism (AFLP) genotype clusters are indicated within a colored box as follows: red, AFLP4/VGI; blue, AFLP10/VGIV; green, AFLP5/VGIII; purple, AFLP7/VGIV; orange, AFLP6/VGII. Colors of the isolate number and locality of isolation refer to their source as follows: red, clinical; blue, animal; green, environmental; black, unknown source. Clinical isolates from Europe that have an autochthonous origin are indicated with a red asterisk, those isolates that were probably acquired outside the European continent are indicated with a blue hash mark. Scale bar indicates number of substitutions per site.

Table 1. Background information of *Cryptococcus gattii* isolates and GenBank accession numbers for the 10-loci multilocus sequence typing data*

Population	Source	Source/Remark	Publication	PMID number
North America	Veterinary	Canine, isolated at 17/01/2007	Hagen et al. Antimicrobial Agents Chemother. 2010;54:5139-5145	29755729
North America	Veterinary	Feline, isolated at 10/02/2006	Hagen et al. Antimicrobial Agents Chemother. 2010;54:5139-5145	29755729
North America	Veterinary	Canine, isolated at 10/02/2006	Hagen et al. Antimicrobial Agents Chemother. 2010;54:5139-5145	29755729
North America	Veterinary	Canine, isolated at 11/03/2006	Hagen et al. Antimicrobial Agents Chemother. 2010;54:5139-5145	29755729
North America	Veterinary	Feline, isolated at 13/04/2006	Hagen et al. Antimicrobial Agents Chemother. 2010;54:5139-5145	29755729
North America	Veterinary	Feline, isolated at 13/04/2006	Hagen et al. Antimicrobial Agents Chemother. 2010;54:5139-5145	29755729
North America	Veterinary	Canine, isolated at 04/04/2006	Hagen et al. Antimicrobial Agents Chemother. 2010;54:5139-5145	29755729
North America	Veterinary	Feline, isolated at 11/07/2006	Hagen et al. Antimicrobial Agents Chemother. 2010;54:5139-5145	29755729
South America	Veterinary	Cheetah (Zoological Park of La Habana, Cuba; Cheetah was imported from South Africa)	Illnait-Zaragozi et al. Mycoses. 2011;54:e889-e892	21668523
North America	Veterinary	Feline, isolated at 29/06/2007	Hagen et al. Antimicrobial Agents Chemother. 2010;54:5139-5145	29755729
North America	Veterinary	Canine, isolated at 12/06/2007	Hagen et al. Antimicrobial Agents Chemother. 2010;54:5139-5145	29755729
North America	Veterinary	Canine, isolated at 30/06/2007	Hagen et al. Antimicrobial Agents Chemother. 2010;54:5139-5145	29755729
North America	Veterinary	Canine, isolated at 29/06/2007	Hagen et al. Antimicrobial Agents Chemother. 2010;54:5139-5145	29755729
North America	Veterinary	Feline, isolated at 15/03/2008	Hagen et al. Antimicrobial Agents Chemother. 2010;54:5139-5145	29755729
North America	Veterinary	Feline, isolated at 10/06/2008	Hagen et al. Antimicrobial Agents Chemother. 2010;54:5139-5145	29755729
Europe	Environmental	*Eucalyptus camaldulensis* leaf (isolated 11/06/1996)	Montagna et al. J Mycol Méd. 1997;7:93-96	n/a
Europe	Environmental	*Eucalyptus camaldulensis* flower (isolated 11/06/1996)	Montagna et al. J Mycol Méd. 1997;7:93-96	n/a
Europe	Environmental	*Eucalyptus camaldulensis* bark (isolated 11/06/1996)	Montagna et al. J Mycol Méd. 1997;7:93-96	n/a
North America	Clinical	Clinical	Ganz et al. J Clin Invest. 1985;76:1427-1435	2997278
Europe	Veterinary	Ostrich (*Struthio camelus*) feather (isolated 11/06/1996)	Montagna et al. J Mycol Méd. 1997;7:93-96	n/a
South America	Environmental	Nest of *Polybia occidentalis* (communal wasp; 1989)	Gezuele et al. Rev Iberoam Micol. 1993;10:5-6.	n/a
Unknown	Clinical	Clinical	Colom et al. Med Mycol. 2012;50:67-73	21521012
Europe	Veterinary	Goat lung, Pescueza outbreak	Colom et al. Med Mycol. 2012;50:67-73	21521012
Europe	Veterinary	Goat lung, Pescueza outbreak	Colom et al. Med Mycol. 2012;50:67-73	21521012
Europe	Veterinary	Goat lung, Serradilla outbreak	Colom et al. Med Mycol. 2012;50:67-73	21521012
Europe	Veterinary	Goat lung, Serradilla outbreak	Colom et al. Med Mycol. 2012;50:67-73	21521012
Europe	Veterinary	Camel (*Camelus bactrianus*) hair (1996)	Montagna et al. J Mycol Méd. 1997;7:93-96	n/a
Europe	Veterinary	Goat liver, Casas de Millan outbreak	Colom et al. Med Mycol. 2012;50:67-73	21521012
Europe	Veterinary	Goat CSF, Casas de Millan outbreak	Colom et al. Med Mycol. 2012;50:67-73	21521012
Europe	Veterinary	Goat lung, Madronera outbreak	Colom et al. Med Mycol. 2012;50:67-73	21521012
Europe	Veterinary	Goat lung, Madronera outbreak	Colom et al. Med Mycol. 2012;50:67-73	21521012

Population	Source	Source/Remark	Publication	PMID number
Europe	Veterinary	Brain tissue of a goat	Colom et al. Med Mycol. 2012;50:67-73	21521012
Europe	Veterinary	Goat lung, Vera outbreak	Colom et al. Med Mycol. 2012;50:67-73	21521012
Europe	Veterinary	Goat lung, Vera outbreak	Colom et al. Med Mycol. 2012;50:67-73	21521012
Europe	Veterinary	Goat lung, Vera outbreak	Colom et al. Med Mycol. 2012;50:67-73	21521012
Europe	Clinical	CSF from human (1995)	Montagna et al. J Mycol Méd. 1997;7:93-96	n/a
Europe	Veterinary	Goat lung, Vera outbreak	Colom et al. Med Mycol. 2012;50:67-73	21521012
Europe	Veterinary	Goose egg (1996)	Montagna et al. J Mycol Méd. 1997;7:93-96	n/a
Europe	Environmental	Small lake with a black swan (*Cygnus atratus*) (1997)	Montagna et al. J Mycol Méd. 1997;7:93-96	n/a
Europe	Environmental	Water in nandu (*Rhea americana*) and emu (*Dromaius novaehollandiae*) cage (1997)	Montagna et al. J Mycol Méd. 1997;7:93-96	n/a
Europe	Environmental	Water in a emu (*Dromaius novaehollandiae*) cage (1997)	Montagna et al. J Mycol Méd. 1997;7:93-96	n/a
Europe	Clinical	CSF from a 37y old male with cirrhosis, had frequently contact with birds/pigeons (2009)	Iatta et al. Mycopathologia. 2012;in press	22057831
Europe	Environmental	Small lake near peacock (*Pavo cristatus*) cage (1997)	Montagna et al. J Mycol Méd. 1997;7:93-96	n/a
Europe	Environmental	Soil from peacock (*Pavo cristatus*) cage (1997)	Montagna et al. J Mycol Méd. 1997;7:93-96	n/a
Europe	Veterinary	Squirrel (*Sciurus* spp.) faeces	Montagna et al. J Mycol Méd. 1997;7:93-96	n/a
Europe	Environmental	Small lake with a black swan (*Cygnus atratus*) (1997)	Montagna et al. J Mycol Méd. 1997;7:93-96	n/a
Europe	Environmental	Water from parrots (Psittaciformes) drinking place (1997)	Montagna et al. J Mycol Méd. 1997;7:93-96	n/a
Asia	Clinical		Taylor et al. J Clin Microbiol. 2002;40:3098-3099	12149391
North America	Clinical	Dead wild Dall's porpoise lumph node autopsy	Kidd et al. Proc Natl Acad Sci USA. 2004;101:17258-17263	15572442
North America	Clinical	Lung tissue from an immunocompetent male	Kidd et al. Proc Natl Acad Sci USA. 2004;101:17258-17263	15572442
North America	Clinical	Dead wild Dall's porpoise mediastinal lymph node	Kidd et al. Proc Natl Acad Sci USA. 2004;101:17258-17263	15572442
North America	Clinical	Bronchial wash from an immunocompetent male	Kidd et al. Proc Natl Acad Sci USA. 2004;101:17258-17263	15572442
North America	Clinical	Bronchial wash from an immunocompetent female	Kidd et al. Proc Natl Acad Sci USA. 2004;101:17258-17263	15572442
North America	Clinical	Bronchial alveolar lavage from an immunocompetent male	Kidd et al. Proc Natl Acad Sci USA. 2004;101:17258-17263	15572442
North America	Clinical	Bronchial alveolar lavage from an immunocompetent female	Kidd et al. Proc Natl Acad Sci USA. 2004;101:17258-17263	15572442
North America	Clinical	Clinical	Kidd et al. Proc Natl Acad Sci USA. 2004;101:17258-17263	15572442
North America	Clinical	CSF from an immunocompetent female	Kidd et al. Proc Natl Acad Sci USA. 2004;101:17258-17263	15572442
North America	Clinical	CSF from an immunocompetent female	Kidd et al. Proc Natl Acad Sci USA. 2004;101:17258-17263	15572442
Africa	Clinical	CSF from a HIV-positive patient	Hagen et al. Antimicrobial Agents Chemother. 2010;54:5139-5145	29755729
North America	Clinical	Human, geographic grid USGS45122-C5D8 (2005)	Byrnes et al. J Infect Dis. 2009;199:1081-1086	19220140
North America	Clinical	Human (2006)	Byrnes et al. J Infect Dis. 2009;199:1081-1086	19220140
North America	Clinical	Unknown	Evans. Proc Soc Exp Biol Med. 1949;71:644-646	18148185
North America	Clinical	CSF from a 45y-old immunocompetent female, traveled to California (1968) and Mexico (1979 and 1980) (1984)	Bottone et al. J Clin Microbiol. 1986;23:186-188	3517042
Europe	Clinical	CSF of HIV-negative human (isolate AV54S, AV54W and IUM01-4731 are subcultures) (2001)	Bovers et al. Fungal Genet Biol. 2008;45:400-421	18261945
Europe	Clinical	CSF of HIV-negative human (isolate AV54S, AV54W and IUM01-4731 are subcultures) (2001)	Bovers et al. Fungal Genet Biol. 2008;45:400-421	18261945

Population	Source	Source/Remark	Publication	PMID number
Europe	Clinical	Skin of HIV negative human (isolate AV55 and IUM00-5363 are subcultures) (2000)	Bovers et al. Fungal Genet Biol. 2008;45:400-421	18261945
Africa	Clinical	CSF from a HIV-positive patient	Hagen et al. Antimicrobial Agents Chemother. 2010;54:5139-5145	29755729
Africa	Clinical	CSF from a HIV-positive patient	Hagen et al. Antimicrobial Agents Chemother. 2010;54:5139-5145	29755729
Africa	Clinical	CSF from a HIV-positive patient	Hagen et al. Antimicrobial Agents Chemother. 2010;54:5139-5145	29755729
Africa	Clinical	CSF of a male patient	Gatti and Eeckels. Ann Soc Belges Med Trop Parasitol Mycol. 1970;50:689-693	5519205
Asia	Clinical	CSF of 30-yr-old immunocompetent nurse with chronic meningitis	Padhye et al. J Med Vet Mycol. 1993;31:165-168	8509953
Asia	Clinical	Swelling below the knee, 17-year-old male (unknown HIV-status)	Padhye et al. J Med Vet Mycol. 1993;31:165-168	8509953
Africa	Clinical	CSF from a HIV-positive patient	Hagen et al. Antimicrobial Agents Chemother. 2010;54:5139-5145	29755729
Africa	Environmental	Tree hollow of an *Eucalyptus* sp. tree in the centre of Cape Town	This study	n/a
Africa	Environmental	Tree hollow of an *Eucalyptus* sp. tree in the centre of Cape Town	This study	n/a
Africa	Environmental	Tree hollow of an *Eucalyptus* sp. tree in the centre of Cape Town	This study	n/a
Africa	Environmental	Tree hollow of an *Eucalyptus* sp. tree in the centre of Cape Town	This study	n/a
Africa	Environmental	Tree hollow of an *Eucalyptus* sp. tree in the centre of Cape Town	This study	n/a
Africa	Environmental	Tree hollow of an *Eucalyptus* sp. tree in the centre of Cape Town	This study	n/a
Europe	Clinical	CSF of 51y HIV negative male who traveled to Vancouver Island (2005)	Lindberg et al. Emerg Infect Dis. 2007;13:179-179	17370544
Africa	Clinical	CSF from patient with unknown HIV-status	This study	n/a
Asia	Clinical	CSF from HIV- 39y female (2001)	Lui et al. QJM. 2006;99:143-151	16504989
Asia	Clinical	CSF from HIV- 40y male (2003)	Lui et al. QJM. 2006;99:143-151	16504989
Asia	Clinical	CSF from HIV- 34y male (1998)	Lui et al. QJM. 2006;99:143-151	16504989
Asia	Clinical	CSF from HIV- 34y male (1998)	Lui et al. QJM. 2006;99:143-151	16504989
Asia	Clinical	CSF from HIV- 34y male (1998)	Lui et al. QJM. 2006;99:143-151	16504989
Asia	Clinical	CSF from HIV- 34y male (1998)	Lui et al. QJM. 2006;99:143-151	16504989
Europe	Clinical	BAL of 26y female with SLE (isolate CBS10608 and IUM98-1969 are subcultures) (1998)	Velegraki et al. Med Mycol. 2001;39:419-422	12054052
Europe	Clinical	CSF of 31y caucasian male (1996)	Velegraki et al. Med Mycol. 2001;39:419-422	12054052
Europe	Clinical	CSF from HIV- 31y male (2003)	This study	n/a
Europe	Clinical	CSF from a 42-year-old female Dutch resident with SLE who visited Vancouver Island (2007)	Hagen et al. Med Mycol. 2010;48:528-531	19824880
North America	Clinical	CSF from a healthy 45-year-old female resident of Alberta who frequently visited Vancouver Island (2006)	Levy et al. Can J Infect Dis Med Microbiol. 2007;18:197-199	18923724
North America	Clinical	CSF from a healthy 45-year-old female resident of Alberta who frequently visited Vancouver Island (2006)	Levy et al. Can J Infect Dis Med Microbiol. 2007;18:197-199	18923724
Australia	Clinical	Human	Katsu et al. FEMS Yeast Res. 2004;4:377-388	14734018
Europe	Clinical	CSF of immunocompetent child (1989)	Guinea et al. Med Mycol. 2010;48:942-948	20297948
Asia	Clinical	Clinical	Hagen et al. Antimicrobial Agents Chemother. 2010;54:5139-5145	29755729
Asia	Clinical	Clinical	Hagen et al. Antimicrobial Agents Chemother. 2010;54:5139-5145	29755729
Europe	Clinical	Tumour like structure, human (1895)	Curtis. C r de la Soc de Biol. 1895;2:715-718	n/a
South America	Veterinary	Sick goat	Diaz et al. Syst Appl Microbiol. 2000;4:535-545	11249024
North America	Veterinary	Cow with mastitis (1952)	Boekhout et al. Int J Syst Bacteriol. 1997;47:432-442	9103633
Europe	Clinical	Isolated from a 27y old 34w pregnant women, fatal case of cryptococcosis (1957)	Janssens and Beetstra. Ned Tijdschr Geneeskd. 1957;101:824-826	13451717

Population	Source	Source/Remark	Publication	PMID number
Unknown	Unknown	Unknown	Boekhout et al. Int J Syst Bacteriol. 1997;47:432-442	9103633
Unknown	Unknown	Unknown	Boekhout et al. Int J Syst Bacteriol. 1997;47:432-442	9103633
Africa	Clinical	CSF of man	Gatti and Eeckels. Ann Soc Belges Med Trop Parasitol Mycol. 1970;50:689-693	5519205
Africa	Clinical	Human	Boekhout et al. Microbiology. 2001;147:891-907	11283285
North America	Clinical	CSF of patient with cryptococcal meningitis	Boekhout et al. Microbiology. 2001;147:891-907	11283285
North America	Clinical	Sputum, immunocompetent human	Boekhout et al. Microbiology. 2001;147:891-907	11283285
North America	Clinical	Human	Boekhout et al. Microbiology. 2001;147:891-907	11283285
North America	Clinical	CSF from a human	Boekhout et al. Microbiology. 2001;147:891-907	11283285
North America	Clinical	CSF of a male patient	Boekhout et al. Microbiology. 2001;147:891-907	11283285
North America	Clinical	Clinical	Diaz et al. Syst Appl Microbiol. 2000;4:535-545	11249024
North America	Clinical	CSF of a male patient	Boekhout et al. Microbiology. 2001;147:891-907	11283285
Asia	Clinical	CSF of a male patient (1978)	Diaz et al. Syst Appl Microbiol. 2000;4:535-545	11249024
Asia	Clinical	CSF of 42y male (1980)	Boekhout et al. Microbiology. 2001;147:891-907	11283285
Australia	Environmental	Bark debris of *Eucalyptus camaldulensis* (river red gum tree)	Boekhout et al. Microbiology. 2001;147:891-907	11283285
Asia	Clinical	CSF of patient with chronic myeloid leukaemia (1984)	Boekhout et al. Microbiology. 2001;147:891-907	11283285
Asia	Clinical	CSF of patient with chronic meningitis (1987)	Boekhout et al. Microbiology. 2001;147:891-907	11283285
Asia	Clinical	CSF of patient with chronic meningitis (1987)	Boekhout et al. Microbiology. 2001;147:891-907	11283285
Australia	Environmental	Seedling of olive, under canopy of *Eucalyptus camaldulensis* (1989)	Boekhout et al. Microbiology. 2001;147:891-907	11283285
Australia	Environmental	Amosphere in hollow *Eucalyptus camaldulensis*	Boekhout et al. Microbiology. 2001;147:891-907	11283285
Australia	Environmental	Bark of *Eucalyptus camaldulensis*	Boekhout et al. Microbiology. 2001;147:891-907	11283285
North America	Environmental	*Eucalyptus camaldulensis* bark debris (1990)	Boekhout et al. Microbiology. 2001;147:891-907	11283285
Unknown	Clinical	Filamentous mutant of CBS7812 (=clinical *C. gattii*)	This study	n/a
Europe	Clinical	CSF from HIV-positive 41y female who emigrated from Zambia (1995)	This study	n/a
Europe	Clinical	CSF from HIV-positive 41y female who emigrated from Zambia (1995)	This study	n/a
South America	Environmental	Nest of *Polybia occidentalis* (communal wasp; 1989)	Gezuele et al. Rev Iberoam Micol. 1993;10:5-6.	n/a
Asia	Clinical	CSF from 28-year-old man	This study	n/a
South America	Environmental	Litter of *Prunus dulcis* (Almond tree)	Boekhout et al. Microbiology. 2001;147:891-907	11283285
South America	Environmental	Litter of *Prunus dulcis* (Almond tree)	Boekhout et al. Microbiology. 2001;147:891-907	11283285
Unknown	Clinical	Infected skin from man	Boekhout et al. Microbiology. 2001;147:891-907	11283285
North America	Clinical	Meningo-encephalitic lesion in man (1924)	Boekhout et al. Microbiology. 2001;147:891-907	11283285
Europe	Clinical	Drainage of brain-abcess of HIV-negative 60-year-old Spanish farmer with diabetes mellitus type 2 (2003)	Colom et al. J Clin Microbiol. 2005;43:3548-3550	16000503
Europe	Clinical	Clinical (isolate CCA242A, CCA242G, CCA242O and CCA242X are subcultures) (2005)	This study	n/a
Europe	Clinical	Clinical (isolate CCA242A, CCA242G, CCA242O and CCA242X are subcultures) (2005)	This study	n/a
Europe	Clinical	Clinical (isolate CCA242L, CCA242N and CCA242T are subcultures) (2005)	This study	n/a
Europe	Clinical	Clinical (isolate CCA242L, CCA242N and CCA242T are subcultures) (2005)	This study	n/a
Europe	Clinical	Clinical (isolate CCA242A, CCA242G, CCA242O and CCA242X are subcultures) (2005)	This study	n/a
Europe	Clinical	Clinical (isolate CCA242L, CCA242N and CCA242T are subcultures) (2005)	This study	n/a
Europe	Clinical	Clinical (isolate CCA242A, CCA242G, CCA242O and CCA242X are subcultures) (2005)	This study	n/a
Europe	Veterinary	Ferret	Colom et al. Med Mycol. 2012;50:67-73	21521012
Europe	Veterinary	Ferret nasal swab (asymptomic carrier)	Colom et al. Med Mycol. 2012;50:67-73	21521012
Europe	Veterinary	Ferret nasal swab (asymptomic carrier)	Colom et al. Med Mycol. 2012;50:67-73	21521012
Europe	Veterinary	Ferret nasal swab (asymptomic carrier)	Colom et al. Med Mycol. 2012;50:67-73	21521012
Europe	Clinical	Human nasal swab (asymptomic carrier) (2010)	Colom et al. Med Mycol. 2012;50:67-73	21521012
Europe	Clinical	Human nasal swab (asymptomic carrier) (2010)	Colom et al. Med Mycol. 2012;50:67-73	21521012

Population	Source	Source/Remark	Publication	PMID number
Europe	Environmental	Bark swab	Colom et al. Med Mycol. 2012;50:67-73	21521012
Europe	Environmental	Bark swab	Colom et al. Med Mycol. 2012;50:67-73	21521012
Europe	Environmental	Bark swab	Colom et al. Med Mycol. 2012;50:67-73	21521012
Europe	Environmental	Detritus swab	Colom et al. Med Mycol. 2012;50:67-73	21521012
Europe	Environmental	Detritus swab	Colom et al. Med Mycol. 2012;50:67-73	21521012
Europe	Environmental	Detritus swab	Colom et al. Med Mycol. 2012;50:67-73	21521012
Europe	Environmental	Bark swab	Colom et al. Med Mycol. 2012;50:67-73	21521012
Europe	Environmental	Bark swab	Colom et al. Med Mycol. 2012;50:67-73	21521012
Europe	Environmental	Detritus swab	Colom et al. Med Mycol. 2012;50:67-73	21521012
Europe	Environmental	Detritus swab	Colom et al. Med Mycol. 2012;50:67-73	21521012
Europe	Clinical	CSF of immunocompromised 66-year-old Spanish male with SLE (2008)	Solla et al. Enferm Infecc Microbiol Clin. 2008;26:395-396	18588823
Australia	Clinical	CSF from human	Bovers et al. Fungal Genet Biol. 2008;45:400-421	18261945
South America	Environmental	Native tree	Baltazar and Ribeiro. Rev Soc Bras Med Trop. 2008;41:449-453	19009184
Asia	Clinical	CSF from a HIV- 33y old Malaysian male	Koh et al. Med Mycol. 2002;40:221-223	12058737
Asia	Clinical	CSF from a HIV- 69y old Singaporean Male	Koh et al. Med Mycol. 2002;40:221-223	12058737
Australia	Environmental		Halliday et al. J Clin Microbiol. 1999;37:2920-2926	10449476
North America	Clinical	Human	Byrnes et al. J Infect Dis. 2009;199:1081-1086	19220140
North America	Veterinary	Veterinary	Byrnes et al. J Infect Dis. 2009;199:1081-1086	19220140
North America	Veterinary	Veterinary	Byrnes et al. J Infect Dis. 2009;199:1081-1086	19220140
North America	Clinical	Human	Byrnes et al. J Infect Dis. 2009;199:1081-1086	19220140
North America	Environmental	Douglas Fir tree #131	Kidd et al. Proc Natl Acad Sci USA. 2004;101:17258-17263	15572442
North America	Environmental	Alder tree #152	Kidd et al. Proc Natl Acad Sci USA. 2004;101:17258-17263	15572442
South America	Clinical	Urine of HIV positive 53y male (1994)	Katsu et al. FEMS Yeast Res. 2004;4:377-388	14734018
South America	Clinical	CSF of an HIV negative human	Barreto de Oliveira et al. J Clin Microbiol. 2004;42:1356-1359	15004118
South America	Environmental	Tree hollow	Barreto de Oliveira et al. J Clin Microbiol. 2004;42:1356-1359	15004118
South America	Environmental	*Eucalyptus* sp. tree	Barreto de Oliveira et al. J Clin Microbiol. 2004;42:1356-1359	15004118
South America	Environmental	*Eucalyptus* sp. tree	Barreto de Oliveira et al. J Clin Microbiol. 2004;42:1356-1359	15004118
South America	Clinical	CSF of an HIV negative human	Barreto de Oliveira et al. J Clin Microbiol. 2004;42:1356-1359	15004118
South America	Clinical	Clinical	Uno et al. Nihon Ishinkin Gakkai Zasshi. 2001;42:127-132	11479533
North America	Clinical	CSF from human (1986)	Hagen et al. Antimicrobial Agents Chemother. 2010;54:5139-5145	29755729
Africa	Clinical	Human CSF (1986)	Hagen et al. Antimicrobial Agents Chemother. 2010;54:5139-5145	29755729
Africa	Clinical	CSF of AIDS patient (1990)	Hagen et al. Antimicrobial Agents Chemother. 2010;54:5139-5145	29755729
Africa	Clinical	CSF of AIDS patient (1990)	Hagen et al. Antimicrobial Agents Chemother.	29755729

Population	Source	Source/Remark	Publication	PMID number
			2010;54:5139-5145	
Africa	Clinical	Blood of AIDS patient (1990)	Hagen et al. Antimicrobial Agents Chemother. 2010;54:5139-5145	29755729
Africa	Clinical	Blood of AIDS patient (1990)	Hagen et al. Antimicrobial Agents Chemother. 2010;54:5139-5145	29755729
Africa	Clinical	CSF of AIDS patient (1991)	Hagen et al. Antimicrobial Agents Chemother. 2010;54:5139-5145	29755729
Africa	Clinical	CSF of AIDS patient (1991)	Hagen et al. Antimicrobial Agents Chemother. 2010;54:5139-5145	29755729
Africa	Clinical	CSF, human (1969)	Hagen et al. Antimicrobial Agents Chemother. 2010;54:5139-5145	29755729
Africa	Clinical	CSF, human (1966)	Hagen et al. Antimicrobial Agents Chemother. 2010;54:5139-5145	29755729
Africa	Clinical	CSF, human (1957)	Hagen et al. Antimicrobial Agents Chemother. 2010;54:5139-5145	29755729
Africa	Clinical	CSF, human (1953)	Hagen et al. Antimicrobial Agents Chemother. 2010;54:5139-5145	29755729
Africa	Clinical	CSF, human (1951)	Hagen et al. Antimicrobial Agents Chemother. 2010;54:5139-5145	29755729
South America	Clinical	Human (1987)	Hagen et al. Antimicrobial Agents Chemother. 2010;54:5139-5145	29755729
South America	Clinical	Human (1987)	Hagen et al. Antimicrobial Agents Chemother. 2010;54:5139-5145	29755729
Europe	Clinical	HIV- patient, immigrant from Mexico (isolate IHEM14941S and IHEM14941W are subcultures) (1987)	Hagen et al. Antimicrobial Agents Chemother. 2010;54:5139-5145	29755729
Europe	Clinical	HIV- patient, immigrant from Mexico (isolate IHEM14941S and IHEM14941W are subcultures) (1987)	Hagen et al. Antimicrobial Agents Chemother. 2010;54:5139-5145	29755729
South America	Clinical	Non-AIDS-patient (1987)	Hagen et al. Antimicrobial Agents Chemother. 2010;54:5139-5145	29755729
South America	Clinical	Human (1988)	Hagen et al. Antimicrobial Agents Chemother. 2010;54:5139-5145	29755729
South America	Clinical	Human (1988)	Hagen et al. Antimicrobial Agents Chemother. 2010;54:5139-5145	29755729
South America	Clinical	AIDS patient (1988)	Hagen et al. Antimicrobial Agents Chemother. 2010;54:5139-5145	29755729
South America	Clinical	Human (1988)	Hagen et al. Antimicrobial Agents Chemother. 2010;54:5139-5145	29755729
South America	Clinical	Human (1988)	Hagen et al. Antimicrobial Agents Chemother. 2010;54:5139-5145	29755729
South America	Clinical	Human (1988)	Hagen et al. Antimicrobial Agents Chemother. 2010;54:5139-5145	29755729
South America	Clinical	AIDS patient (1989)	Hagen et al. Antimicrobial Agents Chemother. 2010;54:5139-5145	29755729
Europe	Clinical	CSF from an Angolese patient (isolate IHEM16633B and IHEM16633S are subcultures) (2000)	Hagen et al. Antimicrobial Agents Chemother. 2010;54:5139-5145	29755729
Europe	Clinical	CSF from an Angolese patient (isolate IHEM16633B and IHEM16633S are subcultures) (2000)	Hagen et al. Antimicrobial Agents Chemother.	29755729

Population	Source	Source/Remark	Publication	PMID number
			2010;54:5139-5145	
Europe	Clinical	CSF from a HIV-positive Rwandese patient (isolate IHEM19725B and IHEM19725S are subcultures) (2003)	Hagen et al. Antimicrobial Agents Chemother. 2010;54:5139-5145	29755729
Europe	Clinical	CSF from a HIV-positive Rwandese patient (isolate IHEM19725B and IHEM19725S are subcultures) (2003)	Hagen et al. Antimicrobial Agents Chemother. 2010;54:5139-5145	29755729
Asia	Clinical	Human (1984)	Hagen et al. Antimicrobial Agents Chemother. 2010;54:5139-5145	29755729
Unknown	Clinical	Clinical	Cogliati et al. Mycoses. 2012;In press	21815945
Europe	Clinical	Clinical (1993)	Fraser et al. Nature. 2005;437:1360-1364	16222245
South America	Clinical	CSF of immunocompetent 28y-old male (1989)	Fraser et al. Nature. 2005;437:1360-1364	16222245
South America	Clinical	CSF from immunocompetent 11y-old female (1986)	Fraser et al. Nature. 2005;437:1360-1364	16222245
Europe	Clinical	Clinical (isolate IP1996/1120-1 and IP1996/1120-2 are from the same patient) (1996)	Fraser et al. Nature. 2005;437:1360-1364	16222245
Europe	Clinical	Clinical (isolate IP1996/1120-1 and IP1996/1120-2 are from the same patient) (1996)	Fraser et al. Nature. 2005;437:1360-1364	16222245
South America	Clinical	CSF from immunocompetent 36y-old male (1997)	Fraser et al. Nature. 2005;437:1360-1364	16222245
Europe	Environmental	Environmental	Fraser et al. Nature. 2005;437:1360-1364	16222245
Europe	Environmental	Environmental	Fraser et al. Nature. 2005;437:1360-1364	16222245
South America	Clinical	BAL from immunocompetent 36y-old male (1997)	Fraser et al. Nature. 2005;437:1360-1364	16222245
South America	Clinical	CSF of 39y-old male (1997)	Fraser et al. Nature. 2005;437:1360-1364	16222245
South America	Clinical	CSF of 17y-old female (1997)	Fraser et al. Nature. 2005;437:1360-1364	16222245
Europe	Clinical	Clinical (isolate IP1998/1037-1 and IP1998/1037-2 are from the same patient) (1998)	Fraser et al. Nature. 2005;437:1360-1364	16222245
Europe	Clinical	Clinical (isolate IP1998/1037-1 and IP1998/1037-2 are from the same patient) (1998)	Fraser et al. Nature. 2005;437:1360-1364	16222245
South America	Clinical	Clinical	Fraser et al. Nature. 2005;437:1360-1364	16222245
Europe	Clinical	BAL of 27y old HIV2-positive female (see isolate IP99/901-2) (1999)	Fraser et al. Nature. 2005;437:1360-1364	16222245
Europe	Clinical	Lung biopsy of HIV2-positive 27y-old female (see IP99/901-1) (1999)	Fraser et al. Nature. 2005;437:1360-1364	16222245
Europe	Clinical	Clinical (2000)	Fraser et al. Nature. 2005;437:1360-1364	16222245
Africa	Clinical	Clinical	Fraser et al. Nature. 2005;437:1360-1364	16222245
Europe	Clinical	CSF of immunocompetent 31y-old male (2003)	Fraser et al. Nature. 2005;437:1360-1364	16222245
Europe	Clinical	CSF of 51y-old male with AIDS (2003)	Fraser et al. Nature. 2005;437:1360-1364	16222245
South America	Clinical	Lung of immunocompetent 64y-old male (2004)	Fraser et al. Nature. 2005;437:1360-1364	16222245
South America	Clinical	Lung of immunocompetent 64y-old female (2004)	Fraser et al. Nature. 2005;437:1360-1364	16222245
Europe	Clinical	Lung of immunocompetent 43y-old female (2005)	Fraser et al. Nature. 2005;437:1360-1364	16222245
Europe	Clinical	Lung of immunocompetent 52y-old female (2006)	This study	n/a
Europe	Clinical	CSF from male (2006)	This study	n/a
Europe	Clinical	Skin of HIV negative human (isolate AV55 and IUM00-5363 are subcultures) (2000)	Bovers et al. Fungal Genet Biol. 2008;45:400-421	18261945
Europe	Clinical	CSF of HIV-negative human (isolate AV54S, AV54W and IUM01-4731 are subcultures) (2001)	Bovers et al. Fungal Genet Biol. 2008;45:400-421	18261945
Asia	Clinical	CSF of HIV negative human	Cogliati et al. Mycoses. 2012;In press	21815945
Europe	Clinical	CSF of HIV negative Brazilian patient (resident in Italy) (1992)	Cogliati et al. Mycoses. 2012;In press	21815945
Asia	Clinical	CSF of HIV negative human	Cogliati et al. Mycoses. 2012;In press	21815945
Asia	Clinical	CSF of HIV negative human	Cogliati et al. Mycoses. 2012;In press	21815945
Europe	Clinical	BAL of 26y female with SLE (isolate CBS10608 and IUM98-1969 are subcultures) (1998)	Velegraki et al. Med Mycol. 2001;39:419-422	12054052
North America	Veterinary	Feline, geographic grid NTS092G/01 (2005)	Hagen et al. Antimicrobial Agents Chemother. 2010;54:5139-5145	29755729
North America	Environmental	Water (2006)	Hagen et al. Antimicrobial Agents Chemother. 2010;54:5139-5145	29755729
North America	Environmental	Water (2006)	Hagen et al. Antimicrobial Agents Chemother. 2010;54:5139-5145	29755729

Population	Source	Source/Remark	Publication	PMID number
North America	Veterinary	Feline (2006)	Hagen et al. Antimicrobial Agents Chemother. 2010;54:5139-5145	29755729
North America	Veterinary	Canine (2006)	Hagen et al. Antimicrobial Agents Chemother. 2010;54:5139-5145	29755729
North America	Veterinary	Feline (2008)	Hagen et al. Antimicrobial Agents Chemother. 2010;54:5139-5145	29755729
North America	Environmental	Air, geographic grid NTS092G/01 (2004)	Hagen et al. Antimicrobial Agents Chemother. 2010;54:5139-5145	29755729
South America	Veterinary	Parrot liver (2000)	Raso et al. Med Mycol. 2004;42:355-362	15473361
Africa	Clinical		Bovers et al. Fungal Genet Biol. 2008;45:400-421	18261945
North America	Clinical	Human (2005)	Hagen et al. Antimicrobial Agents Chemother. 2010;54:5139-5145	29755729
North America	Clinical	Human (2005)	Hagen et al. Antimicrobial Agents Chemother. 2010;54:5139-5145	29755729
Australia	Clinical		Fraser et al. Nature. 2005;437:1360-1364	16222245
Australia	Clinical	Human	Fraser et al. Nature. 2005;437:1360-1364	16222245
North America	Veterinary	Lung tissue of 19y-old adult deceased male wild living Atlantic bottlenose dolphin (*Torsiops truncatus*)	Miller et al. J Clin Microbiol. 2002;40:721-724	11826007
Europe	Clinical	CSF from a 47y old HIV-positive male (1994)	Hagen et al. J Clin Microbiol. 2012;submitted	n/a
Australia	Clinical		Fraser et al. Nature. 2005;437:1360-1364	16222245
Australia	Clinical	Human	Fraser et al. Nature. 2005;437:1360-1364	16222245
Australia	Environmental	*Eucalyptus camaldulensis* (1999)	Fraser et al. Nature. 2005;437:1360-1364	16222245
North America	Environmental	Hollow from Douglas Fir tree #113	Kidd et al. Proc Natl Acad Sci USA. 2004;101:17258-17263	15572442
North America	Environmental	Composite swab 2m W of Alder tree #152	Kidd et al. Proc Natl Acad Sci USA. 2004;101:17258-17263	15572442
North America	Environmental	Retest of Douglas Fir #126 (hollow 2: 1/2)	Kidd et al. Proc Natl Acad Sci USA. 2004;101:17258-17263	15572442
North America	Environmental	Retest of Douglas Fir #126 (hollow 2: 1/2)	Kidd et al. Proc Natl Acad Sci USA. 2004;101:17258-17263	15572442
Australia	Clinical	Human	Fraser et al. Nature. 2005;437:1360-1364	16222245
Australia	Clinical	Human	Fraser et al. Nature. 2005;437:1360-1364	16222245
Australia	Clinical	Human	Fraser et al. Nature. 2005;437:1360-1364	16222245
Europe	Clinical	German male who visited Vancouver Island (2001)	This study	n/a
Europe	Clinical	Clinical (2001)	This study	n/a
Europe	Clinical	Suisse female who visited Vancouver Island (2006)	Georgi et al. Infection. 2009;37:370-373	19390780
Africa	Clinical		Lemmer et al. Med Mycol. 2004;42:135-147	15124867
Africa	Clinical		Lemmer et al. Med Mycol. 2004;42:135-147	15124867
Europe	Clinical	45y-old immunocompetent German resident, lung cryptococcoma (asymptomatic), patient never left Germany (1985)	Schnaberg et al. Internist (Berl). 1988;29:510-515	3049426
Africa	Clinical		This study	n/a
South America	Clinical	34y old immunocompetent female from Brazil who was hospitalized in Switzerland	This study	n/a
Europe	Clinical	24y-old immunocompetent German resident who developed multifocal encephalomyelitis (1997)	Grosse et al. J Neurol Neurosurg Psychiatry. 2001;70:113-116	11118259
Europe	Clinical	Clinical (1997)	Lemmer et al. Med Mycol. 2004;42:135-147	15124867
Europe	Clinical	Clinical (1998)	This study	n/a

Population	Source	Source/Remark	Publication	PMID number
Africa	Clinical		Lemmer et al. Med Mycol. 2004;42:135-147	15124867
Africa	Clinical		Lemmer et al. Med Mycol. 2004;42:135-147	15124867
Africa	Clinical		Lemmer et al. Med Mycol. 2004;42:135-147	15124867
Asia	Clinical	Second isolate of *C. neoformans* variety *shanghaiensis*	Liao et al. Chin Med J. 1983;96:287-290	6413143
Asia	Clinical	Second isolate of *C. neoformans* variety *shanghaiensis*	Liao et al. Chin Med J. 1983;96:287-290	6413143
South America	Clinical	CSF of HIV-negative human	This study	n/a
Australia	Veterinary	Koala	Fraser et al. Nature. 2005;437:1360-1364	16222245
North America	Environmental	Tree hollow	Meyer et al. Med Mycol. 2009;47:561-570	19462334
North America	Environmental	*Eucalyptus citriodora*	Boekhout et al. Microbiology. 2001;147:891-907	11283285
Australia	Clinical	Lung of HIV negative human (1991)	Meyer et al. Med Mycol. 2009;47:561-570	19462334
Australia	Clinical	CSF of HIV negative human (1993)	Meyer et al. Med Mycol. 2009;47:561-570	19462334
Australia	Clinical	Woody debris of *Eucalyptus tericornis* (1993)	Meyer et al. Med Mycol. 2009;47:561-570	19462334
North America	Environmental	Woody debris of *Eucalyptus tericornis*	Boekhout et al. Microbiology. 2001;147:891-907	11283285
North America	Environmental	Woody debris of *Eucalyptus tericornis*	Boekhout et al. Microbiology. 2001;147:891-907	11283285
North America	Environmental	*Eucalyptus citriodora*	Boekhout et al. Microbiology. 2001;147:891-907	11283285
North America	Environmental	Debris of *Eucalyptus* from car park of zoo	Boekhout et al. Microbiology. 2001;147:891-907	11283285
North America	Environmental	Debris of *Eucalyptus* from car park of zoo	Boekhout et al. Microbiology. 2001;147:891-907	11283285
Africa	Veterinary	Cheetah (1994)	Meyer et al. Med Mycol. 2009;47:561-570	19462334

*Alternative isolate numbers, background information regarding the source and origin of the isolate, as well as the reference to the literature are provided. For each isolate the GenBank accession numbers are provided for each of the ten MLST loci, as well as the Sequence Types (STs) for each locus and for the combination of the ten loci. The sequence data for the loci *CAP59*, *GPD1*, IGS1, *LAC1*, *PLB1*, *SOD1* and *URA5* has been included in the recently launched MLST database (http://mlst.mycologylab.org/).

Table 2. Primers used for multilocus sequence typing

	Primer sequences for amplification and sequencing			
Locus	Forward primer sequence	Reversed primer sequence	Optimal T_M	Reference
CAP10	CAP10L-Fwd<br>5′-GTCGTTTTCGCCGATCCTC-3′	CAP10L-Rvd<br>5′-GCCGTAAGACGTGCCCA-3′	60°C	This study
*CAP59**	CAP59L-Fwd<br>5′-GTGAACAAGCTGCGGC-3′	CAP59L-Rvd<br>5′-GGATTCAGTGTGGTGGAAGA-3′	58°C	This study; *1**
GPD1	GPD1L-Fwd<br>5′-GGTTGTCAAGGTTGGAATCAACGG-3′	GPD1L-Rvd<br>5′-GGAGCGGAAATGACGACCTTCTT-3′	61°C	This study
IGS1	IGSF<br>5′-ATCCTTTGCAGACGACTTGA-3′	IGSR<br>5′-GTGATCAGTGCATTGCATGA-3′	61°C	*2*
LAC1	LAC1F<br>5′-AACATGTTCCCTGGGCCTGTG-3′	LAC1R<br>5′-ATGAGAATTGAATCGCCTTGT-3′	50°C	*2*
MPD1	MPD1L-Fwd<br>5′-CCCAGACTGCCGCTGT-3′	MPD1L-Rvd<br>5′-GTGCCGCTAGGCTTCAAGTA-3′	51°C	This study
PLB1	PLB1F	PLB1R	56°C	*2*

Locus	Primer sequences for amplification and sequencing: Forward primer sequence	Reversed primer sequence	Optimal T_M	Reference
	5′-CTTCAGGCGGAGAGAGGTTT-3′	5′-GATTTGGCGTTGGTTTCAGT-3′		
SOD1	SOD1CGF	SOD1CGR	52°C	*2*
	5′-GATCCTCACGCCATTACG-3′	5′-GAATGATGCGCTTAGTTGGA-3′		
TEF1	TEF1L-Fwd	TEF1L-Rvd	62°C	This study
	5′-CTCGGACGGCGAATCGACCAAGAGG-3′	5′-GACGGTCAGACCCGAGAGCACGC-3′		
URA5	URA5F	URA5R	63°C	*2*
	5′-ATGTCCTCCCAAGCCCTCGAC-3′	5′-TTAAGACCTCTGAACACCGTACTC-3′		

*Sequences and optimal annealing temperatures of primer combinations used for amplification and sequencing of each of the ten nuclear loci are provided. The same primers were used to amplify and sequence the obtained amplicons, an exception was made for sequencing of the *CAP59* amplification product, the reversed primer JOHE15438 (5′-CTCTACGTCGAGCAAGTCAAG-3′) was used instead of CAP59L-Rvd due to the presence of the mono-nucleotide-region in front of the primer site.

Table 3. Sequence type diversity for each of the investigated loci and AFLP genotype clusters

	CAP10		*CAP59*		*GPD1*		IGS1		*LAC1*		*MPD1*		*PLB1*		*SOD1*		*TEF1*		*URA5*		MLST Fraser		MLST Meyer		All MLST loci	
Genotype	n_{ST}	D_{ST}	n_{ST}	D_{ST}	n_{ST}	D_{ST}	n_{ST}	D_{ST}	n_{ST}	D_{ST}	n_{ST}	D_{ST}	n_{ST}	D_{ST}	n_{ST}	D_{ST}	n_{ST}	D_{ST}	n_{ST}	D_{ST}	n_{ST}	D_{ST}	n_{ST}	D_{ST}	n_{ST}	D_{ST}
AFLP4 (146)	7	0.499	14	0.609	10	0.504	22	0.820	10	0.540	15	0.667	7	0.514	25	0.776	20	0.506	9	0.627	69	0.908	54	0.926	76	0.943
AFLP5 (22)	3	0.567	3	0.567	5	0.805	6	0.840	8	0.848	4	0.732	7	0.749	4	0.403	8	0.823	7	0.779	21	0.996	16	0.974	17	0.978
AFLP6 (108)	6	0.239	13	0.754	12	0.784	19	0.832	9	0.490	2	0.019	14	0.679	22*	0.809*	4	0.464	11	0.632	34	0.872	45	0.924	44	0.924
AFLP7 (13)	4	0.654	9	0.910	5	0.821	4	0.679	3	0.692	1	0.000	4	0.615	8	0.859	6	0.795	2	0.154	11	0.974	11	0.974	12	0.987
All isolates (291)	19	0.765	40	0.863	32	0.844	52	0.930	31	0.813	25	0.776	30	0.829	59	0.913	34	0.790	30	0.853	136	0.959	127	0.971	150	0.975

*The number of strains, between brackets, for each of the AFLP genotypes showing the overall distribution. Genotype AFLP10/VGIV has been omitted as a separate group from this analysis due to the low number of strains (n = (*2*) that were identical to each other for all ten loci. The number of sequence types (nST) and the diversity (DST) is provided for each locus as well as for the combined set of loci as previously used by Fraser et al. (*1*) and Meyer et al. (*1*) and all loci combined (current study). The study by Fraser et al. (*1*) included the loci CAP10, GPD1, IGS1, LAC1, MPD1, PLB1 and TEF1. The consensus MLST scheme by Meyer et al. (*2*) included the loci CAP59, GPD1, IGS1, LAC1, PLB1, SOD1, and URA5. The asterisk highlights the absence of four strains in the analysis of the SOD1 locus for which no amplicon could be obtained (Technical Appendix Table 1).

References

1. Fraser JA, Giles SS, Wenink EC, Geunes-Boyer SG, Wright JR, Diezmann S, et al. Same-sex mating and the origin of the Vancouver Island *Cryptococcus gattii* outbreak. Nature. 2005;437:1360–4. PubMed http://dx.doi.org/10.1038/nature04220

2. Meyer W, Aanensen DM, Boekhout T, Cogliati M, Diaz MR, Esposto MC, et al. Consensus multi-locus sequence typing scheme for *Cryptococcus neoformans* and *Cryptococcus gattii*. Med Mycol. 2009;47:561–70. PubMed http://dx.doi.org/10.1080/13693780902953886

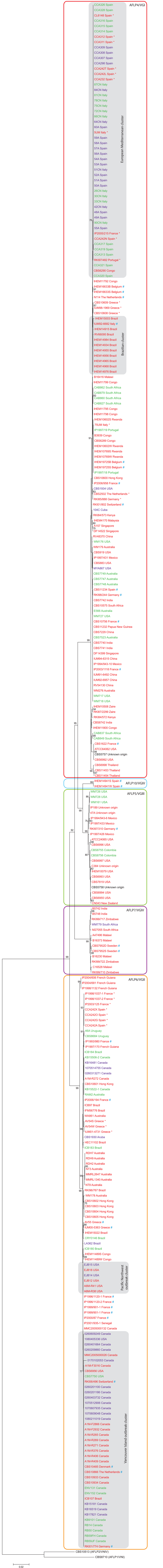
AFLP4/VGI
European Mediterranean cluster
Brazilian cluster
AFLP10/VGIV
AFLP5/VGIII
AFLP7/VGIV
AFLP6/VGII
Pacific Northwest outbreak cluster
Vancouver Island outbreak cluster
CBS10013 (AFLP3/VNIV)
CBS6710 (AFLP1/VNI)
0.02
